# Explaining the Green-Bellied Stink Bugs *Diceraeus* spp. Booming in the Neotropics: Changing from a Secondary to a Key Pest Status

**DOI:** 10.1007/s13744-026-01402-w

**Published:** 2026-05-26

**Authors:** Antônio R. Panizzi, Adeney F. Bueno

**Affiliations:** 1 Embrapa Wheat, Brazilian Agricultural Research Corporation, Passo Fundo, RS 99050-970 Brazil; 2 Embrapa Soybean, Brazilian Agriculture Research Corporation, P.O. Box 4006, Londrina, PR 86001-970 Brazil

**Keywords:** Pentatomidae, pests, feeding, damage, maize, soybean

## Abstract

The green-bellied stink bugs *Diceraeus furcatus* (F.) and *Diceraeus melacanthus* (Dallas) (Hemiptera: Heteroptera: Pentatomidae) were traditionally known as secondary pests of crops in the Neotropics. This situation has changed dramatically, and nowadays, they turned to be key pests of major commodities, such as soybean and maize. They are polyphagous, showing preference for Fabaceae (Leguminosae) and Poaceae (Gramineae). Despite being predominantly seed feeders (soybean bearing pods and cereals bearing seedheads), they also feed on vegetative (seedlings of maize, wheat, and other cultivated spring cereals) plant stages. Feeding activity includes ingestion from xylem vessels, parenchyma cells, and endosperm tissues. Feeding strategies consist on salivary sheath strategy on xylem, laceration (mechanical action by stylets, mostly mandible indentations), and maceration (chemical action of enzymes) causing cells rupture on the parenchyma and seed endosperm. Resulting damage on seedlings of Poaceae includes emission of excess tillering, whitish spots on leaves, wrinkled leaf areas, and tissue necrosis. Feeding during booming causes discolored and deformed seedheads. Fabaceae damage on the seed endosperm shows whitish spots and seed deformation. The uncommon exploration of seedlings by these seed feeding stink bugs, which result in heavy damage, occurs in order to seek for water to get hydrated; acquisition of some nutrients; and achievement of nutrients balance by diluting the concentrated in nutrients sieve from the seed endosperm they usually feed on. Understanding those feeding behaviors allows to establish some important management recommendations such as reducing soybean seed losses and weeds presence at harvest. Seeds on the ground and presence of green weeds are crucial food sources for the green-bellied stink bugs, keeping them active from first to second crop season in the field, favoring their outbreaks.

## Introduction

Stink bugs (Hemiptera: Heteroptera: Pentatomidae) have been recognized as important pests of major (commodities) (Bryant and Reay-Jones 2025) and of minor crops (Adamič-Zamljen et al. 2025) including vegetables and fruit trees all over the world for a long time (Schaefer and Panizzi 2000). Recently, their increased importance has been discussed globally, highlighting main pest species, and their bioecology and sustainable management strategies on different geographical areas of the world (Bueno and Panizzi 2024; Panizzi et al. 2026).

In the Neotropical region, because of favorable environmental conditions during most of the year, stink bugs develop and reproduce in a continuum, which make them very successful (Vieira et al. 2025). Many crops are cultivated year-round in several cropping systems forming an abundant and always present food availability (“green bridge”) (Garcia et al. 2018). This provides an ideal and highly favorable environment for their development and reproduction, which ends up on frequent eruptions of stink bugs populations, causing severe damage to crops.

Among the several species of stink bugs that have evolved very successfully on this scenario, the green-bellied stink bugs, *Diceraeus furcatus* (F.) and *Diceraeus melacanthus* (Dallas) are classical examples. For a long time, they were considered secondary pests, particularly *D. furcatus* attacking soybean in some countries of the Neotropics, such as Brazil (Panizzi et al. 1977), Argentina (Rizzo 1976), and Uruguay (Abbate et al. 2022). *Diceraeus melacanthus* was not even recorded as a pest species, until it was reported damaging maize seedlings by the middle 90s (Ávila and Panizzi 1995).

With time passing and some radical changes in cropping systems been incorporated on modern agriculture, the status of several pests changed dramatically (Ma et al. 2025), including stink bugs (Bueno and Panizzi 2024). For example, the southern green stink bug, *Nezara viridula* (L.), for a long time considered the number one pest of soybean in the Neotropics, decreased radically to very low numbers; in contrast, other species also change their pest status and turned key pests (Panizzi and Lucini 2016).

The main factors believed to influence the dynamics of pest populations of stink bugs in the Neotropics have been presented and discussed (Panizzi et al. 2022). However, their feeding habits and strategies that helped to allow their increase in abundance and successfully achievement as major pests have never been analyzed in detail. In this review, we will focus on the dramatic change in the importance of the green-bellied stink bugs *D. furcatus* and *D. melacanthus*. We will analyze their relation with associated and host plants, their preference (although polyphagous) for species of cultivated plants in the families Fabaceae and Poaceae, and how they feed on these plants in the non-reproductive and the reproductive stages. Their feeding strategy will be present and discussed as a very sophisticated process in order to achieve a balanced diet considering nutrients and water content, seeking to achieve their best reproductive performance. This is essential knowledge not only to understand those species success but also to further propose effective management strategies.

## Associated and Host Plants

*Diceraeus furcatus*, as the majority of phytophagous stink bugs, is polyphagous, and, on many plants where they are found on, they do not develop or reproduce. They use them for maintenance (providing some nutrients and water) or for shelter; which are called associated plants. In a smaller number of plants, stink bugs do achieve nymph development and adult reproduction, and these are called host plants. The further characterization of these two types of plants related to stink bugs can be found in Panizzi and Lucini (2022).

*Diceraeus furcatus* is more commonly found in the Neotropics in areas cultivated with commodities (e.g., soybean, maize, wheat) usually below the tropic of Capricorn, in general, with lower temperatures than northern areas (see distributional map in Jacobi et al. 2022). For example, in Brazil, it is recorded more often in the cooler southern states of the Southern region (see distributional map in Panizzi et al. 2015).

In a literature review of plants that *D. furcatus* have been found on (32 species), the majority of them (78%—25 species) were associated, and the minority (22%—7 species) were hosts; plants belonged to 13 families, which confirm their polyphagous feeding habits (Smaniotto 2015; Smaniotto and Panizzi 2015).

*Diceraeus melacanthus*, similar to the previous species, is polyphagous. Its distribution in the Neotropics is also general. However, at least in Brazil is known to occur mainly in warmer areas of the Central-West region, associated with widely cultivated commodities (see distributional map in Panizzi et al. 2015).

Data from the literature indicates *D. melacanthus* reported on 29 species of plants from 10 different families. The majority of them (83%, 24 species) are associated plants, and host plants include the remaining ones (17%, 5 species) (Smaniotto 2015; Smaniotto and Panizzi 2015).

More recently, several species of weed plants, from different families, have been found, either associated or serving as hosts to both *D. furcatus* and *D. melacanthus*. Despite some studies showing nymphs developing and adult reproducing on weeds, their role influencing the phenology of these stink bugs populations have been largely overseen and underestimated (Panizzi and Lucini 2022).

## Preference for Fabaceae and Poaceae Host Plants

**Fig. 1:**
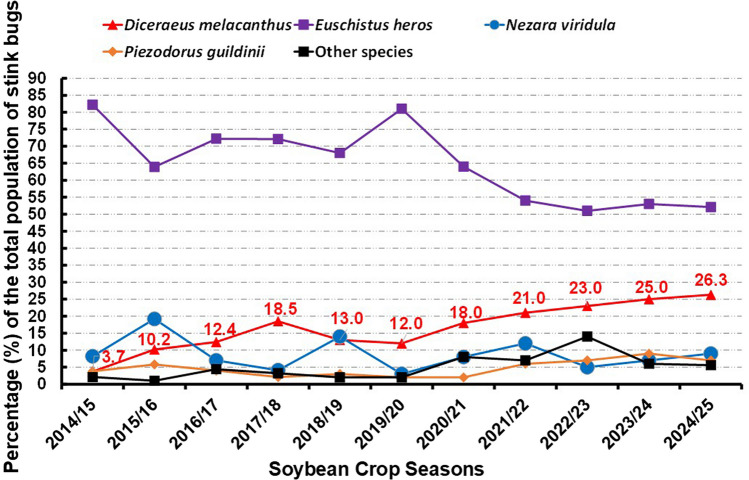
Distribution of stink bug species in soybean fields (Paraná state, South Brazil) from 2014/2015 to 2024/2025 crop seasons. Note the steady and constant increase in the populations of *Diceareus melacanthus*, in particular in the last five crop seasons (adapted from Bueno et al. 2023)

**Fig. 2:**
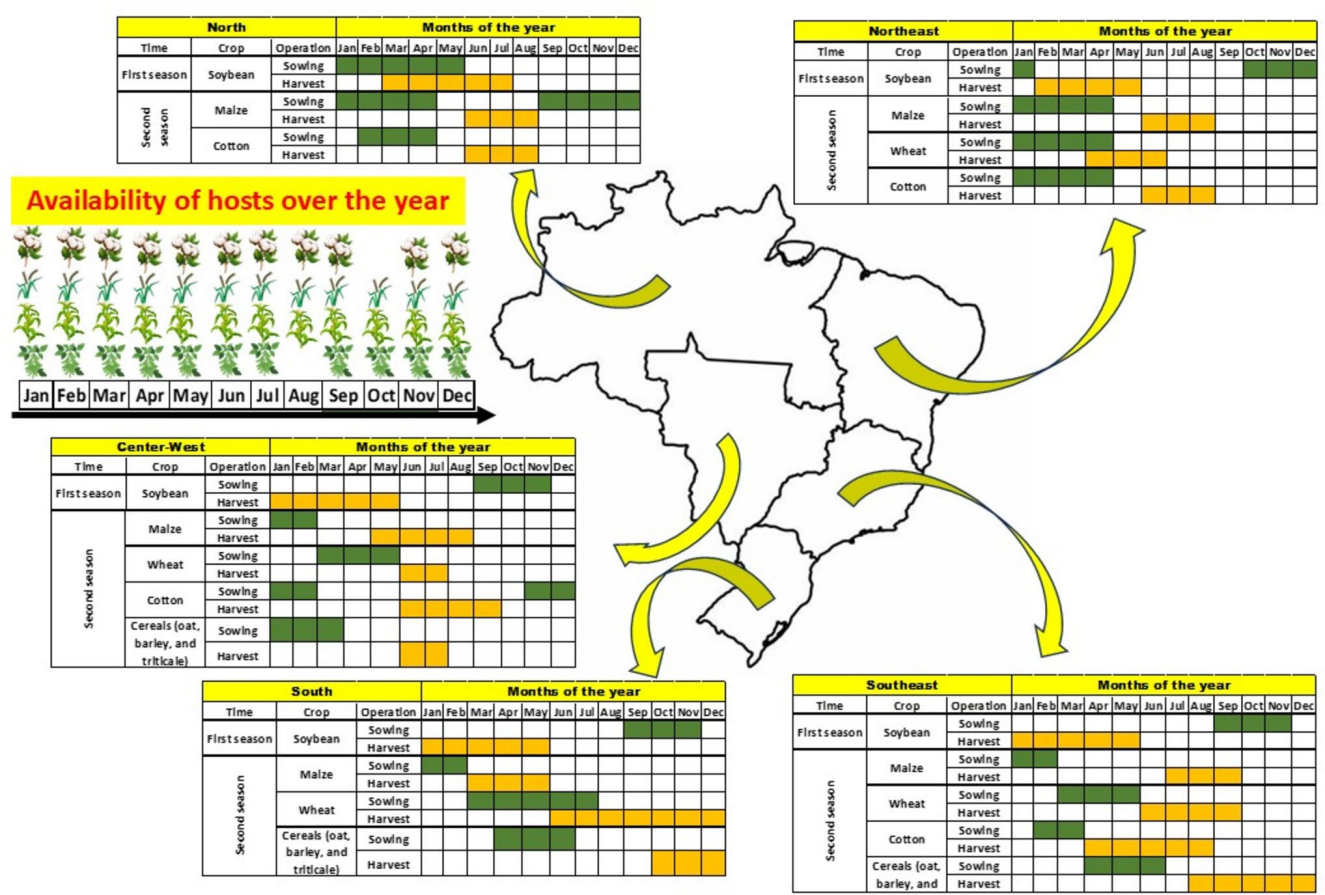
Intensive modern-day agriculture including different commodities constantly offering food resources to pests in the Neotropics, with crops cultivated in sequence along the year forming the so-called green bridge

The green-bellied stink bugs, *D. furcatus* and *D. melacanthus*, were considered secondary pests in the 1970s and 1980s, reaching only 1% of the total species of pest stink bugs in the Neotropics (Brazil). From the 1990s on, *D. melacanthus* grew in abundance reaching 11%, becoming the second most abundant stink bug pests in the Neotropics (Panizzi et al. 2022; Saldanha et al. 2024). For instance, in the state of Paraná, South of Brazil, *D. melacanthus* has been increasing its abundance on soybean from 3.7 (2014/2015) to 26.3% (2024/2025 crop season) (Fig. 1). It is a significant increase in abundancy in this time frame (ca. 611%) in soybean, as a result of continuous food availability (Fig. 2), allowing this pest to actually become the most important pest on maize (Queiroz et al. 2022).

*Diceraeus furcatus* has been traditionally associated with soybean plants during the reproductive period (Panizzi et al. 1977) and, eventually, colonizing wheat plants (Panizzi et al. 2016a, b). In Argentina, this species is also reported to damage soybean (Luna and Iannone 2013) and seedlings of maize (Bonivardo et al. 2025).

Among the many plants the two species of green-bellied stink bugs are found on, colonizing them or not, species belonging to the families Fabaceae and Poaceae are the most preferred. Considering the first plant family (Fabaceae), *D. furcatus* is reported to occur on 10 species, with only three species, soybean, alfalfa, and common bean, serving as hosts. *Diceraeus melacanthus* is reported to occur on four species, three of them serving as host plants, soybean, and the non-cultivated lance leaf crotalaria and hairy indigo (Table 1).

**Table 1:** *Diceraeus furcatus* and *Diceraeus melacanthus* registered on different species of Fabaceae serving as hosts or associated plants

Scientific name	Common name	Host plant	Associated plant
***Diceraeus furcatus***			
* Glycine max* (L.) Merr	Soybean	X	-
* Lotus corniculatus* L.	Bird’s foot trefoil	-	X
* Lupinus albus* L.	White lupin	-	X
* Macroptilium atropurpureum* Urb	Purple bush-bean	-	X
* Medicago sativa* L.	Alfalfa	X	-
* Phaseolus vulgaris* L.	Common bean	X	-
* Pisum sativum* L.	Common pea	-	X
* Rhynchosia corylifolia* Mart	Least snout-bean	-	X
* Vicia* spp.	Vetches	-	X
* Vigna sinensis* L.	Cowpea	-	X
***Diceraeus melacanthus***			
* Crotalaria pallida* Ait	Smooth crotalaria	-	X
* Crotalaria lanceolata* E. Mey	Lanceleaf crotalaria	X	-
* Glycine max* (L.) Merr	Soybean	X	-
* Indigofera hirsuta* L.	Hairy indigo	X	-

Based on information from references in Smaniotto and Panizzi (2015) and Panizzi et al. (2015)

On Poaceae plants, *D. furcatus* is mentioned to occur on five species, and only one (wheat) serving as host plant. *Diceraeus melacanthus* is reported on 10 species, including cultivated crops and grasses. Similar to what was observed for *D. furcatus*, wheat is the only species reported as host (Table 2). Interesting to note that in studies conducted in the laboratory with *D. furcatus*, nymphs completed development and adults reproduced feeding on reproductive structures (seedheads) of wheat, black oat, barley, rye, and triticale (Panizzi et al. 2018).

**Table 2:** *Diceraeus furcatus* and *Diceraeus melacanthus* registered on different species of Poaceae serving as hosts or associated plants

Scientific name	Common name	Host plant	Associated plant
***Diceraeus furcatus***			
* Avena sativa* L.	White oat	-	X
* Avena strigosa* Schreb	Black oat	-	X
* Lolium multiflorum* Lam	Ryegrass	-	X
* Triticum aestivum* L.	Wheat	X	-
* Zea mays* L.	Maize	-	X
***Diceraeus melacanthus***			
* Avena strigosa* Schreb	Black oat	-	X
* Brachiaria decumbens* Stapf	Signal grass	-	X
* Brachiaria plantaginea* (Link) Hitchc	Alexander grass	-	X
* Cenchrus echinatus* L.	Southern sandbur	-	X
* Chloris gayana* Kunth	Rhodes grass	-	X
* Eleusine indica* (L.) Gaertn	Goosegrass	-	X
* Megathyrsus maximus* (Jacq.) BKSimon & SWL Jacobs	Guinea grass	-	X
* Triticum aestivum* L.	Wheat	X	-
* Triticosecale semisecale* (Mackey) K. Hammer & Filat	Triticale	-	X
* Zea mays* L.	Maize	-	X

Based on information from references in Smaniotto and Panizzi (2015) and Panizzi et al. (2015)

Apparently, these stink bug species are unable to recognize these plants in nature as suitable sources of nutrients to allow reproduction. This is reinforced by the fact that nymphs developing on seedheads of these plants are rarely seen in the field, although adults may more often be seeing feeding on seedheads. Clearly, additional studies are needed to fully explain their interactions with those plants. Laboratory studies demonstrated that *D. melacanthus* completed development on soybean pods, wheat seedheads, and on maize seeds (Chocorosqui and Panizzi 2008).

### Feeding on Vegetative and on Reproductive Plant Structures

#### Feeding on stem of seedling

Field observations indicate that the green-bellied stink bugs are commonly found feeding on the stem of seedling of the cultivated Poaceae, mostly wheat and maize. On wheat/maize seedlings, both species *D. furcatus* and *D. melacanthus* use two strategies: salivary sheath strategy, on xylem vessels; and laceration and maceration (cells rupturing activities) on the parenchyma cells (Lucini and Panizzi 2017a, b). These authors fully characterize these feeding strategies using the electrical penetration graph (EPG) technique by relating the electrical waveforms produced with the specific feeding sites in the plants.

**Fig. 3:**
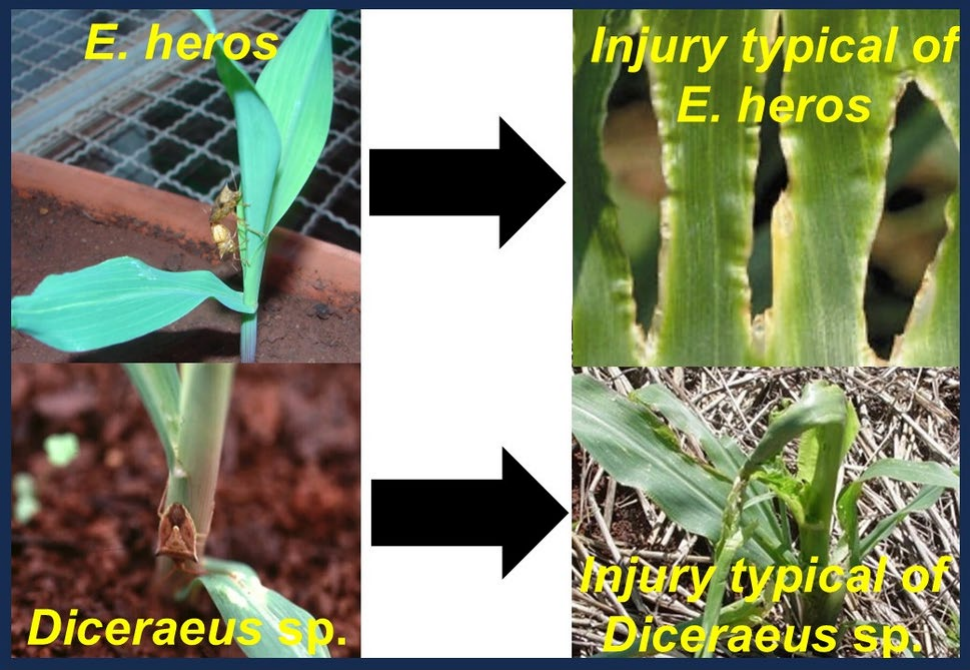
Different feeding behavior and feeding damage of adult green-bellied stink bugs, *Diceraeus* spp. and the Neotropical brown stink bug, *Euschistus heros* on maize seedlings

During xylem feeding, a salivary sheath is completely formed, which serve to anchor the stylets (mandibles + maxillae) and help in the ingestion process. Interesting to note that during this type of feeding, in general, stink bugs are found in the downward position (Fig. 3). This has been speculated that this position might facilitate the intake of the xylem sap that moves upward, i.e., from roots to leaves. On the contrary, in the upward position, the flow of the xylem sap runs away, making the ingestion process harder (schematic representation of stink bug body position and xylem sap flow is detailed in Panizzi and Lucini 2019). Similarly, the brown-winged stink bug *Edessa meditabunda* (F.), which feeds on stems of soybean, has been also observed to feed in downward position (Panizzi and Machado Neto 1992).

In addition, *Diceraeus* spp. are usually found close to the ground while the Neotropical brown stink bug, *Euschistus heros* (F.), the most abundant stink bug in the soybean-maize system in the Neotropics, which also feeds on maize seedlings, is more frequently found on the leaves (Fig. 3). It is also speculated that those different feeding habits are responsible for *Diceraeus* spp. being a key pest in maize, while *E. heros*, despite commonly found over the maize seedlings, is not (it causes only holes on the leaves—Fig. 3). This injury is usually supported by maize plants without any economic yield reduction (Gomes et al. 2020). That makes a great difference for maize management. While *D. melacanthus* needs to be controlled during maize emergence with two or more adults per linear meter, *E. heros* do not requires control, even with population of six adults per meter (Gomes et al. 2020). Green-bellied stink bug feeding increases the chances of damaging shoot apical meristem, while *E. heros* feeding results in injuries limited to reduced foliar photosynthesis (Fig. 3). Therefore, *Diceraeus* spp. damage capacity will depend on maize stalk diameter (Santos et al. 2025). The most susceptible growth stages include V1–V5 or V1–V7 (Silva et al 2019) which vary accordingly to maize cultivars (Castilhos et al. 2026).

During feeding on parenchyma cells via cell rupture, stink bugs secrete an incomplete stylet sheath using gel saliva using laceration and maceration feeding strategies. During laceration, stylets move in the vegetal tissue fast and deep, tearing the tissue mostly by the indentation of the mandibles (mechanical cell destruction). During maceration, stylets move in a slow pattern to spread the saliva rich on enzymes to degrade the cells contents. Sucking insects use laceration and maceration in separate, while stink bugs use both simultaneously, which can be considered and evolution in the feeding process of sucking insects (Lucini and Panizzi 2018).

#### Feeding on seed

The feeding activity of the green-bellied stink bugs during the reproductive phase of their hosts, usually occurs on immature seeds. As previously discussed, they use the laceration and maceration strategies to obtain nutrients from seeds, which are rich  in nutrients. Studies conducted with different species of pest stink bugs [e.g., *Nezara viridula* (Panizzi and Slansky 1991); *D. melacanthus* (Chocorosqui and Panizzi 2008)] have demonstrated that they need to feed on seeds to be able to complete nymph development and to reproduce. Although nymphs can develop partially, and adults may reproduce to a very low degree on vegetative plant structures, the obtention of nutrients from seeds is mandatory for those pests to complete their lifecycle. The green-bellied stink bugs, feeding on seedlings of maize and wheat, perfectly illustrate that. Nymphs are rarely found on these seedlings, and egg masses, indicating reproduction, are also rarely found on them, in spite of the heavy feeding activity and resulting tissue damaging (see discussion ahead).

Despite the growing incidence of the green-bellied stink bugs on soybean, in particular of *D. melacanthus*, not much information is available on their damaged caused to seeds. However, it may be similar to what is caused by other species of pentatomids, i.e., seed deformation of early developing seeds and whitish spots on fully developed seeds (Queiroz et al. 2022). Therefore, the economic thresholds (ETs) to control stink bugs on soybean was defined for the total number of stink bugs per row meter (adding all species) as two stink bugs (from third-instar on to adults)/soybean row meter from R3 to R6 soybean stages (Panizzi 1980; Bueno et al. 2026) .

Similarly, the feeding on wheat seed by *D. furcatus* has been illustrated, showing whitish spots in the endosperm as seed contents were damaged mechanically (stylets serration) and chemically (enzymes activity) (Lucini and Panizzi 2017a). Also, seeds do not develop and seedheads appear discolored and malformed when bugs feed on them or earlier during booting (early stage of seedheads formation) (Panizzi et al. 2016a, b). Similar damage is caused by *D. melacanthus* feeding during wheat seed development (Chocorosqui and Panizzi 2004). However, different from soybean, in general, there is no need to control *D. furcatus* on wheat, unless numbers are ≥ 8 bugs per m^2^ during reproductive period (Panizzi et al. 2016a, b).

Different from soybean, maize, and wheat, ETs for green-bellied stink bugs on other cereals have not been scientifically established. Future studies must address this issue to precisely help farmers avoiding unnecessary use of chemical insecticides.

## Nutritional Balance and Hydration by Feeding on Vegetative Plant Structures

Feeding by the green-bellied stink bugs on major crops (soybean, maize, and wheat) as here presented indicates that this has been very favorable to both species, proved by the great increase in their populations in the field (Queiroz et al. 2025). The cultivation of these crops in sequence in the Neotropics, forming the so-called green-bridge, seems to be the most important condition favoring the increase of green-bellied stink bugs populations. Moreover, the presence of mature seeds on the ground after crop harvesting (Queiroz et al. 2022), and the ability of these stink bugs to stay on the ground sheltered under crop residues, waiting for favorable environmental conditions to feed on them, further explain their success.

Known by feeding preferably on reproductive structures of soybean (pods) and wheat (seedheads), the heavy damage caused by the green-bellied stink bugs to seedlings of maize, wheat, and other spring cultivated cereals (e.g., barley, rye, oat, and others) might come as a surprise (see discussion of damages ahead). Why is this occurring in such a high level, forcing growers to apply insecticides early in the cereal cultivated in the second season and/or having to treat their seeds with insecticides to prevent failure of those crops?

The possible answer to that can be explained by analyzing their nutritional needs. As the soybean crop is harvested at the end of summer, a great portion of stink bugs stay on the ground. This happens because there is food available there, i.e., fallen mature seeds (from harvest losses) ready to be explored and different weeds. For instance, an average of 4 to 6% of soybean has been considered to be lost during the harvest process in the Neotropics (Goldsmith et al. 2015; Arends-Kuenning et al. 2022). Stink bugs are able to feed on mature seeds, introducing their stylets penetrating the seedcoat (Queiroz et al. 2022). Also, seeds on the ground become softer as they get in contact with the moisture on the soil from crop residues, which facilitates seed penetration. As a new crop is seeded (usually maize or wheat), stink bugs start feeding on the developing seedlings. This behavior has three main reasons: first, obtain of water to get hydrated, which is very important to survivorship; second, acquisition of some nutrients which are present in the developing seedlings, in particular immediately after germination, when nutrients storage in the seed cotyledons start to circulate in the young plantlet; and third, achievement of nutrients balance, i.e., the right dilution of nutrients in the body. Feeding on vegetative tissue also occurs when stink bugs feed on soybean seeds on the plants, by switching from the seed endosperm to the xylem of leaves and stems to dilute the nutrients concentration (Panizzi and Lucini 2019).

## Heavy Feeding Damage to Seedling Plants

Feeding on seedlings of Poaceae plants cause whitish spots on leaves, they may show wrinkled areas resembling plant virus damage and tissue necrosis. Later during booting, feeding result in discolored and deformed seedheads (Panizzi et al. 2015, 2016a, b). On maize, feeding on the stem close to the soil, cause seedling plants to wilt and eventually die (Ávila and Panizzi 1995).

An additional damage to wheat and maize seedlings is the emission of excess tillering as referred by the authors above. As the main stem is damaged, plants react by emitting several tillers in order to compensate the failure of the main stem. On spring cereals, mostly on wheat, this is a common plant response to stink bug injury, however in many cases passing overseen, as plantlets grow close to one another. Finally, leaves of spring cereals damaged can die and turned filiform, also, in general, not recognize as a result of stink bugs feeding activity.

## Final Considerations

In modern agriculture under way in the Neotropics, several new cultural practices have been introduced. They include the wide spread adoption of no or minimum tillage, which increased the demand for herbicides to manage the weeds; the introduction of genetically modified (GM) crops; and use of early maturity soybean cultivars to allow multiple cultivations of different crops in the same field during the year. These practices cause disruption on wild and cultivated plants availability in the new landscape; and new pressures are added that affect the role of pests and natural enemies in general (Panizzi et al. 2022).

In this new scenario (Fig. 2), some species of stink bugs, once considered minor pests, change their status and became key pests. This is what happened to the two species of green-bellied stink bugs, *D. furcatus* and *D. melacanthus*. They fast adapted to explore the rich source of nutrients on the soil (mature seeds fallen after harvesting) which remain available for relatively long time, in conjunction with exploration of young plants (including weeds and volunteer plants). This feeding adaptation allowed those stink bugs to reach water and nutrients abundantly available at the same place and time. The combination of these two components plus the fact that crop residues after harvest also offer shelter and protection against both, biotic (natural enemies) and abiotic (solar radiation, rain, peak in temperature) factors, complete the favorable framework for those stink bugs (*D. furcatus* and *D. melacanthus*) success.

Therefore, for the effective management of green-bellied stink bugs in these intensive agroecosystems of multiple crops cultivated in the same field, important recommendations are reduction of seed losses during harvest [around 4–6% or 128–192 kg of soybean (885 to 1168 volunteer soybean plants) per hectare in Brazil (Barbosa et al. 2020)]; and reduction of weeds presence at harvesting. Any crop management aiming these two recommendations will certainly reduce green-bellied stink bug populations, towards reaching their effective management.

## Data Availability

It is not applicable since it is a review article.
